# Distribution of the Mosquito Communities (Diptera: Culicidae) in Oviposition Traps Introduced into the Atlantic Forest in the State of Rio de Janeiro, Brazil

**DOI:** 10.1089/vbz.2017.2222

**Published:** 2018-04-01

**Authors:** Shayenne Olsson Freitas Silva, Cecilia Ferreira de Mello, Ronaldo Figueiró, Daniele de Aguiar Maia, Jeronimo Alencar

**Affiliations:** ^1^ Diptera Laboratory, Oswaldo Cruz Institute (Fiocruz), Rio de Janeiro, Brazil.; ^2^ Postgraduate Program in Tropical Medicine, Oswaldo Cruz Institute (Fiocruz), Rio de Janeiro, Brazil.; ^3^ Postgraduate Program in Animal Biology, Institute of Biology, Federal Rural University of Rio de Janeiro, Rio de Janeiro, Brazil.; ^4^ Environmental Biotechnology Laboratory, Fundação Centro Universitário Estadual da Zona Oeste (UEZO), Rio de Janeiro, Brazil.; ^5^ Centro Universitário de Volta Redonda (UniFOA), Volta Redonda, Brazil.; ^6^ Universidade Castelo Branco (UCB), Rio de Janeiro, Brazil.

**Keywords:** *Aedes albopictus*, climatic variables, Culicidae, eggs, *Haemagogus janthinomys*, *Haemagogus leucocelaenus*

## Abstract

The Atlantic Rainforest of South America is one of the major biodiversity hotspots of the world and serves as a place of residence for a wide variety of Culicidae species. Mosquito studies in the natural environment are of considerable importance because of their role in transmitting pathogens to both humans and other vertebrates. Community diversity can have significant effects on the risk of their disease transmission. The objective of this study was to understand the distribution of mosquito communities using oviposition traps in a region of the Atlantic Forest. Sampling was carried out in Bom Retiro Private Natural Reserve (RPPNBR), located in Casimiro de Abreu, Rio de Janeiro, using oviposition traps, which were set in the forest environment, from October 2015 to December 2016. The canonical correspondence analysis was used to assess the influence of the climatic variables (precipitation, maximum dew point, and direction) throughout the seasons on the population density of the mosquito species. The results showed that population density was directly influenced by climatic variables, which acted as a limiting factor for the mosquito species studied. The climatic variables that were significantly correlated with the density of the mosquito species were precipitation, maximum dew point, and direction. *Haemagogus janthinomys* was positively correlated with the three climatic variables, whereas *Haemagogus leucocelaenus* was positively correlated with precipitation and maximum dew point, and negatively correlated with direction.

## Introduction

The Atlantic Forest comprises a set of forest formations that have a rich diversity of mosquito species with considerable spatial variability. The topographic complexity of this environment allows the existence of a broad spectrum of microclimates and environmental conditions that influence the availability and sustainability of mosquito habitats (Alencar et al. 2011, Marques et al. 2012, Correa et al. 2014).

Climate change can affect biodiversity at different levels by accelerating the metabolism of some individuals, and affecting the food chains and ecological interactions of populations and communities (Hughes 2000). One of the most critical issues related to climate change is its impact on disease vectors (Chaves and Koenraadt 2010). According to Alencar et al. (2011), the activity level of different mosquito species is directly influenced by the climatic variables, such as temperature and air humidity. Understanding their biodiversity, richness, abundance, and response to anthropological activities is essential for predicting changes in their populations (Alencar et al. 2016).

Community diversity can significantly increase the risk of pathogen transmission from vectors to humans (Keesing et al. 2006). Nevertheless, a greater variety of hosts in a more diverse community with less competent hosts may reduce the incidence of the disease in the focal host by a “dilution effect.” This hypothesis was demonstrated by Johnson and Thieltges (2010) in his observational study on the transmission of *Schistosoma mansoni* Sambon, 1907, where he found that increasing the diversity of a community substantially reduces the transmission of the parasite. This connection between species diversity and disease transmission by vectors is based on zooprophylaxis, using animals that attract hematophagous insects away from humans.

However, it has been suggested that high diversity can increase the risk of transmission when there is a greater supply of alternative hosts. The amount and activity of the insect vectors increase and these hosts function as alternative sources of infection. Hence, it is important to know the vector community, and eventually their hosts, to perform an overall assessment of the transmission risk (Holt and Pickering 1985, Norman et al. 1999, Gilbert et al. 2001, Schmidt and Ostfeld 2001, Saul 2003, Dobson 2004).

One of the surveillance methods for these vectors includes the use of oviposition traps. It is a sensitive method for mosquito detection (Resende et al. 2013) taking into consideration the species that do oviposit in ovitraps, which can generate indices that aid in the early detection of new infestations (Gomes 1998), and is economically and operationally viable (Braga and Valle 2007). This trap assists in the determination of geographic dispersion, density, frequency, and seasonality (Juliano 1998, Glasser and Gomes 2000, Passos et al. 2003). Some characteristics observed in the adult insects are largely a product of their larval environment (Braks et al. 2004), which may affect their vectorial competence (ability to become infected), and consequently, their ability to transmit the pathogen (Hardy and Monath 1988). Oviposition traps have been successfully used to obtain the eggs of *Haemagogus leucocelaenus* Dyar and Shannon, 1924 (Medeiros 2009), *Haemagogus equinus* Theobald, 1903 (Chadee and Tikasingh 1990), and *Haemagogus janthinomys* Dyar, 1921 (Alencar et al. 2004).

Mosquitoes of the genus *Haemagogus* Williston, 1896, and *Sabethes* Robineau-Desvoidy, 1827, are the most epidemiologically important species involved in the transmission of wild-type yellow fever virus, thereby acting as biological vectors in the forest areas of the Americas (Arnell 1973). The *Haemagogus* species are wild, with diurnal habits, and are most active in the tree canopies, however, some of these species show a tendency of adaptation to human environments (Marcondes and Alencar 2010). According to the Ministry of Health data from December 2016 to May 31, 2017, there were 3240 reports of suspected cases of wild-type yellow fever, of which 519 (16%) remained under investigation, 792 (24.5%) were confirmed, and 1929 (59.5%) discarded. About 79 cases were reported to the State Health Department, Rio de Janeiro, of which 27 were confirmed and 55 discarded from January to August 2017. Of the 27 confirmed cases, 8 had their origin point from the municipality of Casimiro de Abreu and one from Silva Jardim.

This study observed the distribution of effective or potential vector species of wild-type yellow fever virus that colonize the oviposition traps and analyzed the influence of climatic variables (precipitation, maximum dew point, and direction) on the vector distribution during different seasons of the year in an area of the Atlantic Forest with confirmed wild-type yellow fever cases.

## Materials and Methods

### Ethics statement

The permanent license for collecting, capturing, and transporting zoological material from the RPPNBR was granted by the Environment and Agriculture Secretariat (SISBIO) with number: 34911-1, dated June 14, 2012, across all the national territory.

### Area of study

**FIG. 1.**
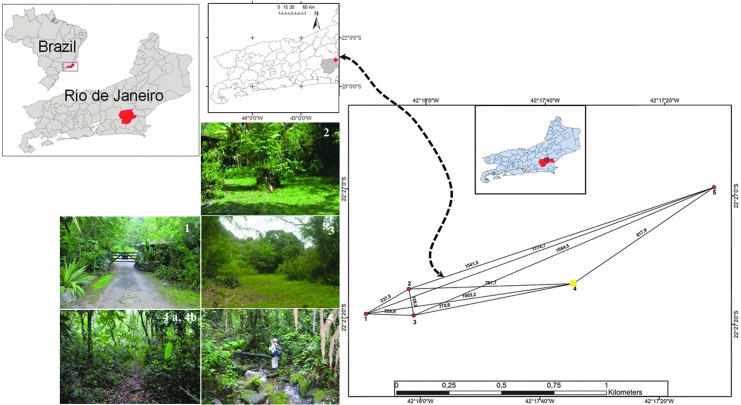
Sampling sites in the RPPNBR, located in the city of Casimiro de Abreu, state of Rio de Janeiro. RPPNBR, Bom Retiro Private Natural Reserve.

The RPPNBR, Casimiro de Abreu Municipality, Rio de Janeiro State, ∼140 km from Rio de Janeiro, has an area largely covered by the typical Atlantic Forest vegetation that persists for most of the year under the control of the Tropical Mass (MTA) originated from the Tropical Atlantic Antictone. The region experiences average annual temperatures ranging from 18 to 24°C, due to the marked solar radiation of tropical latitudes, and strong relative humidity due to intense sea evaporation. Because of its own characteristics, the dominance of this air mass maintains the stability of the weather, although interference from the Polar Fronts or discontinuities, and Tropical Instability Lines (Schobbenhaus et al. 1995), which promote weather instability, might occur during the year. Such disturbed currents are largely responsible for the annual rainfall, particularly along with the contribution of summer rains that generate a predominantly tropical humid climate (Takizawa 1995). The area of the RPPNBR in the Sao Joao River Basin is located in the intertropical zone (low latitudes), with intense solar radiation and great influence of the Atlantic Ocean (Cunha 1995), producing a tropical wet climate. Geographical coordinates of the sampling sites were obtained using the Garmin GPS map 60 CS GPS. Maps were prepared in ArcView10 and edited in Adobe Photoshop CS5 and CorelDraw X5. The sampling sites were as follows: Sites in RPPNBR, state of Rio de Janeiro, Brazil; Site 1—RPPNBR entrance, the secondary forest under the direct influence of the river and waterfalls (22°27′19.4″ S, 42°18′09.5″ W); Site 2—located near the reserve management, an anthropogenic environment and composed of forests in the advanced stage of regeneration (22°27′15.4″ S, 42°18′02.4″ W); Site 3—the entrance to the forest, with vegetation in the advanced stage of regeneration (22°27′19.5″ S, 42°18′01.5″ W); Site 4a and 4b—forest with original vegetation and some parts showing bamboo monoculture (22°27′14.1″ S, 42°17′34.9″ W); Site 5—contains large areas with forests similar to the original biocenotic structure (22°26′58.7″ S 42°17′11.6″ W) (Fig. 1).

Monitoring was performed using oviposition traps that consisted of a black pot, with a 1 L capacity without a lid, and four plywood pallets (Eucatex^®^ plates), measuring 2.5 × 14 cm, and fixed vertically inside the trap by “CLIPS” (Alencar et al. 2016). Natural water and remains of leaves and animals found on the forest soil were added into the trap to generate an ecosystem similar to the natural one. The oviposition traps were installed on trees by using a fishing sinker (diameter ∼4 cm) and secured using a nylon rope, and 24 ovitraps were set at two heights (ground level and 2.50 meters) in the forest and monitored through October 2015 to December 2016, weekly by replacing the panels with new ones; 12 ovitraps were set for each height. All the paddles were sequentially numbered and placed in a humid chamber and sent to the Diptera Laboratory of the Oswaldo Cruz Institute.

In the laboratory, the positive paddles (containing eggs) were separated, had their eggs counted, and immersed in clear trays containing MiliQ^®^ water. The collected eggs were placed to hatch in a controlled experimental environment, in a thermoperiod and a photoperiod regulated at 28°C ± 1°C, relative humidity of 75% to 90%, and photoperiod of 10 h. The specimens were kept alive for specific determination in adulthood, by direct observation of the morphological characters evidenced by the stereomicroscopic microscope (Zeiss^®^) and consultation with the respective descriptions/diagnoses of the spp, using dichotomous keys developed by Consoli and Oliveira (1994), Forattini (2002), and Marcondes and Alencar (2010). Abbreviations for the generic and subgeneric names were assigned in accordance to Reinert (2001). After species determination, all the specimens were incorporated into the Entomological Collection of the Oswaldo Cruz Institute, Fiocruz, under the title “Culicidae Mata Atlântica.”

The data were analyzed to assess the ecological relationship between the Culicidae populations and the climatic variables of the study area. The Canoco 4.5 program was used to evaluate and compare the differences in the composition of the mosquito communities and the relationship between the population density and the climatic variables (precipitation, maximum dew point, and direction) (Ter Braak and Simaluaer 2002). The canonical correspondence analysis was performed to evaluate the correlation structure between the mosquito community and the climatic variables. The Monte Carlo simulation generates random data matrices to prove the presence/absence of the effects of the variables.

## Results

During the collection period from October 2015 to December 2016, a total of 7186 eggs were collected and 1206 specimens of Culicidae were identified, representing three genera and five species: *H. (Conopostegus) leucocelaenus* Dyar & Shannon, 1924: 992 specimens; *H. (Haemagogus) janthinomys* Dyar, 1921: 63 specimens; *Aedes (Stegomyia) albopictu*s Skuse, 1894: 103 specimens; *Aedes (Stegomyia) aegypti* Linnaeus, 1752: 42 specimens; *Culex (Carrollia) iridescens* Lutz, 1905: 4 specimens; and *Limatus durhamii* Theobald, 1901: 2 specimens (Table 1).

**Table 1.** The Percentage and Number of Species Collected from Each of the Collection Points Located in Bom Retiro Private Natural Reserve, State of Rio de Janeiro, Brazil, from October 2015 to October 2016

*Species*	*Site 1*	*Site 2*	*Site 3*	*Site 4 A*	*Site 4 B*	*Site 5*	*Total*	*%*
*Haemagogus janthinomys*	0	0	0	1	5	57	63	5.22
*Haemagogus leucocelaenus*	2	0	0	137	633	220	992	82.26
*Aedes albopictus*	44	55	3	0	1	0	103	8.54
*Aedes aegypti*	0	0	0	0	42	0	42	3.48
*Culex iridescens*	0	0	0	0	4	0	4	0.33
*Limatus durhamii*	0	0	0	2	0	0	2	0.17
Total	46	55	3	140	685	277	1206	100

*Monthly variation*—maximum abundance of *H. leucocelaenus*, was observed in December 2015 and November 2016, whereas *H. janthinomys* was more frequent in February and November 2016. *A. albopictus* was found most frequently in October and November 2015, whereas *A. aegypti* and *C. iridescens* were more abundant in October 2015 and November 2015, respectively (Table 2).

**Table 2.** Relative Abundance and Percentage of Mosquito Species Collected Using Oviposition Traps at the Bom Retiro Private Natural Reserve, State of Rio de Janeiro, Brazil, from October 2015 to October 2016

*RPPNBR/No. of species per month*							
*Species Period*	A. albopictus	A. aegypti	H. leucocelaenus	H janthinomys	C. iridescens	Limatus durhamii	*Total*
2015							
October	42	42	67	4	0	0	155
%	3.48	3.48	5.56	0.33	0.00	0.00	10.55
November	54	0	82	2	4	0	142
%	4.48	0.00	6.80	0.17	0.33	0	11.77
December	6	0	219	2	0	0	227
%	0.50	0.00	18.16	0.17	0.00	0.00	18.82
2016							
February	0	0	132	41	0	0	173
%	0.00	0.00	10.95	3.40	0.00	0.00	14.34
March	0	0	14	0	0	0	15
%	0.00	0.00	1.16	0.00	0.00	0.00	1.24
April	9	0	22	0	0	0	31
%	0.75	0.00	1.82	0.00	0.00	0.00	2.57
May	0	0	0	0	0	0	0
%	0	0	0	0.00	0.00	0.00	0.00
June	19	0	0	0	0	0	19
%	1.58	0.00	0.00	0.00	0.00	0.00	1.58
July	0	0	0	0	0	0	0
%	0	0	0	0	0	0	0
August	0	0	9	0	0	0	9
%	0.00	0.00	0.75	0.00	0.00	0.00	0.75
September	0	0	26	1	0	0	26
%	0.00	0.00	2.16	0.08	0.00	0.00	2.16
October	0	0	31	1	0	0	31
%	0.00	0.00	2.57	0.08	0.00	0.00	2.57
November	25	0	333	18	0	0	376
%	2.07	0.00	27.61	1.49	0.00	0.00	31.18
December	0	0	0	0	0	0	0
%	0.00	0.00	0.00	0.00	0.00	0.00	0.00
Total	155	42	935	70	4	0	1206
%	1.85	3.48	77.53	5.80	0.33	0.00	100.00

RPPNBR, Bom Retiro Private Natural Reserve.

**FIG. 2.**
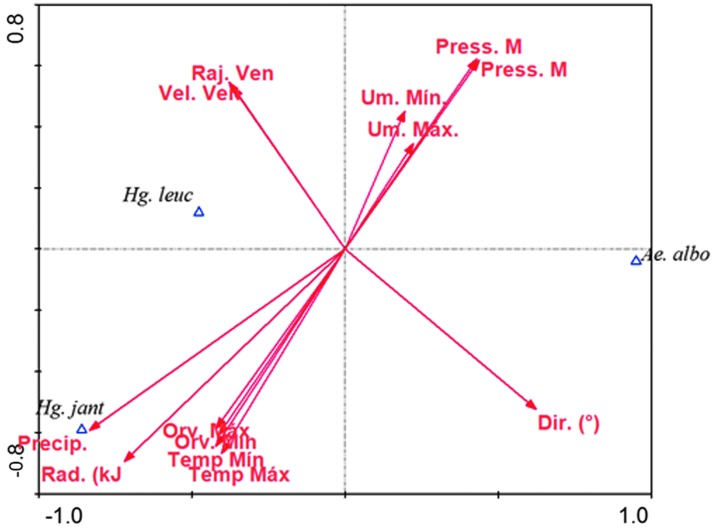
Direction and proximity of the climatic variables. The *red* vectors indicate the direction and proximity of the climatic variables in relation to each species studied. The greater the proximity of these vectors with the species (in *blue triangle* representative of the respective populations), the stronger the interaction between the two. Dir. (^o^) (Direction), Orv. Max (Maximum dew point), Orv. Min (Minimum dew point), Temp Min (Minimum temperature), Temp Max (Maximum temperature), Precip. (Precipitation or Rainfall), Rad. (Radiation), Raj. Ven. (Wind Gust), Vel. Ven. (Wind speed), Press. M (Maximum pressure), Press Min. (Minimum pressure), Um. Mín. (Minimum humidity), Um. Max (Maximum humidity).

The climatic variables that were significantly correlated with mosquito densities were precipitation (*p* = 0.0080), maximum dew point (*p* = 0.0348), and direction (*p* = 0.0346). *H. janthinomys* individuals were positively correlated with the three climatic variables, whereas, *H. leucocelaenus* individuals showed positive correlation with precipitation and maximum dew point, and were negatively correlated with direction (Fig. 2).

**FIG. 3.**
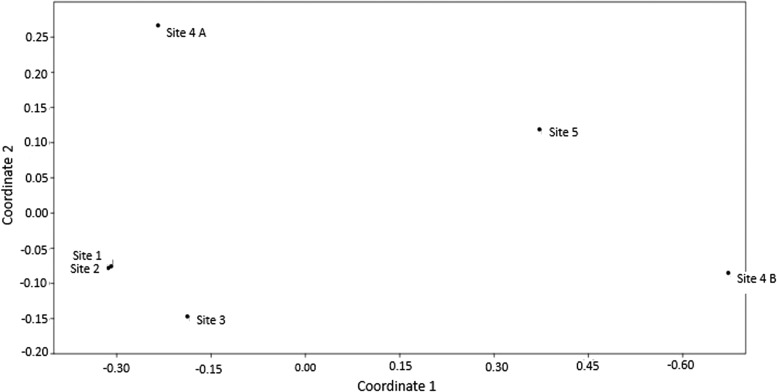
Assessment of the significant differences among the collected sites (Site 1, Site 2, Site 3, Site 4 A, Site 4 B, and Site 5).

Based on the number of the collected mosquito eggs, the results revealed a high level of population density of vectors in summer and spring, comprising 2002 and 1102 eggs in February and November 2016 (Table 3). A simple linear regression was performed using the software R, version 3.4.1, with a confidence index of 95% and *p* < 0.05 to analyze the correlation between the number of eggs and rainfall, using the number of eggs as the discrete variable and rainfall as the continuous variable (Table 4). The regression analysis revealed that the number of mosquito eggs was significantly associated with rainfall (*p* = 0.003561) (Fig. 3).

**Table 3.** Number of Hatched Eggs and Nonhatched Eggs, Collected from October 2015 to November 2016 at the Bom Retiro Private Natural Reserve, State of Rio de Janeiro, Brazil

*Months/years*	*Hatched eggs*	*Nonhatched eggs*	*Total*
October/2015	36	408	444
November/2015	73	492	565
December/2015	105	998	1103
February/2016	818	1184	2002
March/2016	14	43	57
April/2016	81	281	362
May/2016	0	1	1
June/2016	0	21	21
July/2016	69	42	111
August/2016	168	283	451
September/2016	280	268	548
October/2016	117	302	419
November/2016	238	864	1102
Total	1999	5187	7186

**Table 4.** Number of Eggs Collected at the Bom Retiro Private Natural Reserve, State of Rio de Janeiro, from February to October 2016, and Rainfall in mm According to the Data from the National Institute of Meteorology

*Months/years*	*Eggs*	*Rainfall (mm)*
February/2016	2002	0.56
March/2016	57	0.29
April/2016	362	0.06
May/2016	1	0.06
June/2016	21	0.07
July/2016	111	0.01
August/2016	451	0.02
September/2016	548	0.07
October/2016	419	0.11
November/2016	1102	0.39

**FIG. 4.**
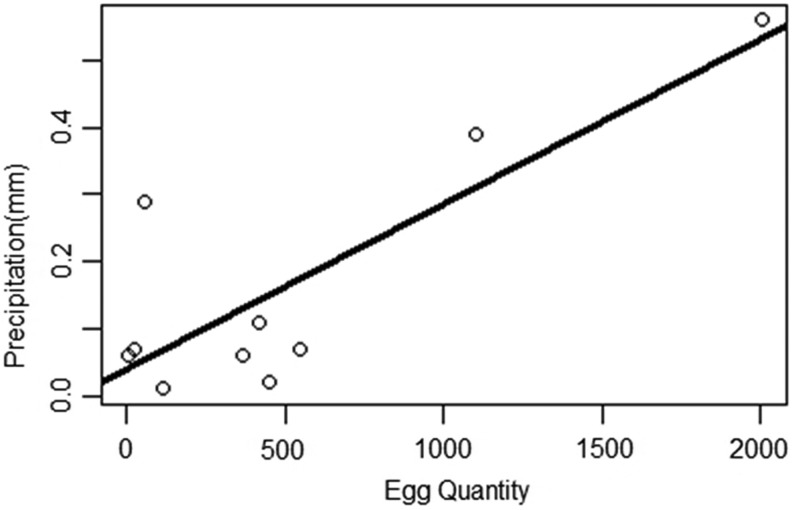
Number of eggs collected at RPPNBR, state of Rio de Janeiro, Brazil, from February to October 2016, and rainfall in mm (INMET). INMET, National Institute of Meteorology.

To understand the similarities and differences between the sampling units, a nonmetric multidimensional scale was used to represent the original position of the mosquito communities in the multidimensional space as accurately as possible using a small number of dimensions that can be easily plotted and visualized. Figure 4 shows the level of similarity and differences among the sampling points of collection based on their proximity to each other. Coordinates 1 and 2 assist in the allocation of these points in a Cartesian plane.

The most distinct collection sites observed were 4A and 4B and most similar were 1 and 2. The sampling sites 4A and 3 were similar in relation to coordinate 1; however, they were distinct from coordinate 2. Sites 3 and 4 B were similar in relation to coordinate 2; however, they were very different from coordinate 1 (Fig. 4).

## Discussion

Understanding the biodiversity of mosquito species in the Atlantic Forest is fundamental for the prediction of possible changes in their populations. The mosquito fauna present in this environment has great biodiversity, including potential vectors of the yellow fever virus and other arboviruses. Mosquitoes of the genera *Haemagogus* and *Sabethes* spp. are the main vectors of the wild-type yellow fever virus in the forest areas of the Americas, and are of major importance in the transmission of this arbovirus (Vasconcelos et al. 2003).

Most of the species found in this study are known to be vectors of several agents considered pathogenic to humans. *H. janthinomys* stands out as the main vector of the wild-type yellow fever virus in the Americas, as well as being a vector of other arboviruses, such as Mayaro and Ilheus (Vasconcelos et al. 2003). *H. leucocelaenus* is a vector of wild-type yellow fever virus in Brazil (Arnell 1973). *A. aegypti* and *A. albopictus* are known vectors of dengue virus (WHO 2017). *A. aegypti* is also known to transmit other viral diseases, such as yellow fever, chikungunya (Powell and Tabachnick 2013), and Zika (Marchette et al. 1969, Diallo et al. 2014). *Culex* can transmit pathogens responsible for causing encephalitis, lymphatic filariasis, and heartworm disease (Service 1993).

Alencar et al. (2015) conducted a study in the Guapiaçu Ecological Reserve, Rio de Janeiro, Brazil, and found that the largest number of specimens was observed in April and December. Similarly in this study, a peak was observed in the number of *A. albopictus* and *H. leucocelaenus* in April and December. The highest number of individuals of *H. janthinomys* was observed in February 2016. This result is similar to that obtained by Pinto et al. (2009) in a study conducted in the National Forest of Caxiuanã, Pará, Brazil, who reported an increased abundance of *H. janthinomys* in the same month.

In this study, *H. leucocelaenus* showed a positive correlation with precipitation and maximum dew point, indicating an influence of these abiotic factors on its behavior and subsequent increase in its population. Similar results were reported by Resende et al. (2013) who reported a positive and significant correlation between rainfall and monthly frequency of *H. leucocelaenus*.

Alencar et al. (2011) reported that the populations of *H. janthinomys* analyzed were significantly influenced by rainfall, leading to a change in the activity rhythm, thereby increasing the population density in the rainy seasons. This study also showed that *H. janthinomys* individuals were favored by the three climatic variables: precipitation, maximum dew point, and direction.

Marteis et al. (2017) found that a high relative air humidity index guaranteed the maintenance of natural breeding sites in the wild environments and promoted the formation of larval habitats, consequently influencing the population density of adults. These observations are in agreement with the results of this study, considering that both *H. leucocelaenus* and *H. janthinomys* species were positively correlated with maximum dew point. Patz et al. (2003) reported that the increase in rainfall influences the breeding behavior of the vectors, consequently favoring population growth. The same trend was also observed in both *H. leucocelaenus* and *H. janthinomys* species that had a positive correlation with precipitation in this study.

Furthermore, the fact that many human communities in different Brazilian regions do extensive work and/or leisure activities in the nocturnal twilight period is worthy of special attention. These activities increase the chances of their encounter with the vectors of yellow fever virus. In addition, such activities can also increase the confirmed yellow fever cases in nonhuman primates in the region surrounding the RPPNBR, which highlights the importance for conducting entomological surveillance in this area.

## Conclusions

Knowledge regarding the distribution of vector species is crucial for the improvement and maintenance of operations that promote the surveillance and control of these arthropods and with evidence of active sylvatic yellow fever virus transmission next to the natural reserve studied here, the abundance of the mosquito vector for this disease in Brazil requires active surveillance on the emergence of the virus in neighboring communities (Forshey et al. 2010).
